# Behaviour change, weight loss and remission of Type 2 diabetes: a community‐based prospective cohort study

**DOI:** 10.1111/dme.14122

**Published:** 2019-09-26

**Authors:** H. Dambha‐Miller, A. J. Day, J. Strelitz, G. Irving, S. J. Griffin

**Affiliations:** ^1^ Primary Care Unit Department of Public Health and Primary Care University of Cambridge School of Clinical Medicine, Institute of Public Health Cambridge UK; ^2^ MRC Epidemiology Unit University of Cambridge School of Clinical Medicine, Institute of Metabolic Science Cambridge UK; ^3^ Primary Care and Population Sciences University of Southampton Southampton UK

## Abstract

**Aim:**

To quantify the association between behaviour change and weight loss after diagnosis of Type 2 diabetes, and the likelihood of remission of diabetes at 5‐year follow‐up.

**Method:**

We conducted a prospective cohort study in 867 people with newly diagnosed diabetes aged 40–69 years from the ADDITION‐Cambridge trial. Participants were identified via stepwise screening between 2002 and 2006, and underwent assessment of weight change, physical activity (EPAQ2 questionnaire), diet (plasma vitamin C and self‐report), and alcohol consumption (self‐report) at baseline and 1 year after diagnosis. Remission was examined at 5 years after diabetes diagnosis via HbA_1c_ level. We constructed log binomial regression models to quantify the association between change in behaviour and weight over both the first year after diagnosis and the subsequent 1–5 years, as well as remission at 5‐year follow‐up.

**Results:**

Diabetes remission was achieved in 257 participants (30%) at 5‐year follow‐up. Compared with people who maintained the same weight, those who achieved ≥ 10% weight loss in the first year after diagnosis had a significantly higher likelihood of remission [risk ratio 1.77 (95% CI 1.32 to 2.38; *p*<0.01)]. In the subsequent 1–5 years, achieving ≥10% weight loss was also associated with remission [risk ratio 2.43 (95% CI 1.78 to 3.31); *p*<0.01].

**Conclusion:**

In a population‐based sample of adults with screen‐detected Type 2 diabetes, weight loss of ≥10% early in the disease trajectory was associated with a doubling of the likelihood of remission at 5 years. This was achieved without intensive lifestyle interventions or extreme calorie restrictions. Greater attention should be paid to enabling people to achieve weight loss following diagnosis of Type 2 diabetes.

## What's new?

- Biochemical remission of Type 2 diabetes in the absence of pharmacological or surgical intervention has been shown to be achievable.
- This has been previously demonstrated in short‐term studies and only in selected populations through intensive weight loss programmes.
- We found that weight loss of ≥10% in the first few years after diagnosis was strongly associated with remission of Type 2 diabetes at 5 years.
- This was achieved without intensive lifestyle interventions or extreme calorie restrictions.
- Our findings should inform discussions with people who have newly diagnosed Type 2 diabetes as a motivation towards remission of the disease without restrictive and sometimes unachievable calorie restrictions.

**What's new?**

- Biochemical remission of Type 2 diabetes in the absence of pharmacological or surgical intervention has been shown to be achievable.
- This has been previously demonstrated in short‐term studies and only in selected populations through intensive weight loss programmes.
- We found that weight loss of ≥10% in the first few years after diagnosis was strongly associated with remission of Type 2 diabetes at 5 years.
- This was achieved without intensive lifestyle interventions or extreme calorie restrictions.
- Our findings should inform discussions with people who have newly diagnosed Type 2 diabetes as a motivation towards remission of the disease without restrictive and sometimes unachievable calorie restrictions.

## Introduction

Type 2 diabetes affects 400 million people globally and has been characterized as a lifelong progressive disease 1, 2; however, biochemical remission or ‘cure’, defined as a level of glycaemia below the diagnostic threshold (HbA_1c_ < 48 mmol/mol or 6.5%) 3 in the absence of pharmacological or surgical interventions, is achievable through significant calorie restriction and weight loss 4, 5. Although there are varying definitions of remission in the literature, we have adhered to that based on HbA_1c_ levels in line with UK and US national guidance 3. Intensive low‐calorie diet (total energy intake of 624–700 kcal/day) for 8 weeks was associated with remission in 87% of people with recently diagnosed diabetes (<4 years) and in 50% of people with longstanding disease (>8 years)^4^. Similarly, the Action for Health in Diabetes (Look AHEAD) study included an intensive 4‐year programme, designed to increase physical activity and reduce initial weight by ≥7% 6. Participants had a median diabetes duration of 5 years and, in the first year after the intervention, 11.5% achieved partial or complete remission compared to only 2.0% in the usual care group. In the DIRECT trial, participants who had been diagnosed with diabetes in the previous 6 years underwent an intensive intervention, including withdrawal of diabetes and blood pressure medication, diet replacement of 825–853 kcal/day through a formula diet for 3–5 months, stepped food reintroduction (2–8 weeks), and structured support for long‐term weight loss maintenance 7. Remission was achieved in 46% of the intervention group. Collectively, these studies support the hypothesis that healthy behaviour change and weight loss can result in remission of diabetes.

In all these studies, however, selected participants were recruited to intensive weight loss interventions. Evidence is therefore required from representative population‐based samples undergoing less intensive interventions that are more feasible and potentially scalable to the wider population. Furthermore, most studies have either examined remission in the short term or amongst people who have lived with diabetes for a few years, or both 6, 7. It is unclear if behaviour change and weight loss early in the disease trajectory could lead to long‐term remission. This is important as there could be a window of opportunity following diagnosis when people might be more receptive to interventions concerning weight loss. Using data from the ADDITION‐Cambridge population‐based study of screening for Type 2 diabetes, we quantified the association between behaviour change and weight loss in the year after diagnosis and the subsequent 4 years, in relation to the likelihood of remission of diabetes at 5‐year follow‐up.

## Methods

### Study design and setting

We conducted a cohort study analysis using prospectively collected data from the ADDITION‐Cambridge trial (registered as ISRCTN86769081). This is a pragmatic, parallel group cluster randomized controlled trial conducted among 49 general practices in the East of England. Individuals aged 40–69 years who were not known to have diabetes and had a Cambridge Diabetes Risk Score ≥ 0.17 (corresponding to the top 25% of participants’ risk distribution) were invited to attend a stepwise screening programme for Type 2 diabetes, including initial random capillary glucose and HbA_1c_ testing followed by a fasting capillary glucose and a confirmatory oral glucose tolerance test 8, 9, 10. Diagnosis of Type 2 diabetes was based on the 1999 WHO criteria 11. Exclusion criteria were current pregnancy, lactation, psychiatric disease that prevented informed consent, or an illness with a likely prognosis of <1 year at the time of diabetes diagnosis. All 867 participants identified by screening agreed to participate and were randomized at a practice level into either the intervention group (multifactorial treatment) or control group (routine care). In the routine care group, practices were advised to follow current UK national guidelines for diabetes management 12. Intensive treatment comprised more frequent consultations, including a 30‐min annual review, in addition to three 10‐min consultations with a general practitioner and nurse, provision of educational materials and guidelines, and practice‐based academic detailing sessions, encouraging earlier use of medication to improve control of risk factors, with a local diabetologist and a general practitioner opinion leader who described treatment algorithms and targets. A detailed description of the trial has been reported in previous publications 8, 9 **.**

### Exposure and outcome measurements

All measures were taken at baseline, 1‐ and 5‐year follow‐up**.** Physical activity was assessed by self‐report using the validated EPIC Physical Activity Questionnaire (EPAQ‐2) 13. Dietary intake was assessed by self‐report using a validated semi‐quantitative food frequency questionnaire, which enabled estimation of daily intake of total energy, and fat as a percentage of energy and fibre intake 14, 15. Alcohol intake and smoking status (categorized as never smoked, ex‐smoker or current smoker) were assessed by self‐report via questionnaire. Clinical and biochemical measures were collected by trained staff following standardized protocols, as previously described 12, 17. Blood pressure was calculated as the mean of three measurements using an automatic sphygmomanometer. Body weight and height were measured in light clothing and without shoes using a scale (SECA) and a fixed rigid stadiometer, respectively. Venous blood samples were collected for analysis of lipid and HbA_1c_ levels.

Remission was defined as an HbA_1c_ level < 48 mmol/mol (6.5%) in the absence of any diabetes medication or bariatric surgery. Information on medication use and a history of bariatric surgery was self‐reported and we also reviewed patient electronic general practice records. At baseline, none of the participants included in this cohort were on any hypoglycaemic agents. We then followed up the whole cohort for 5 years, regardless of whether they were subsequently commenced on medications. Those who went into remission were by definition not on hypoglycaemic medication at the 5‐year follow‐up.

### Statistical analysis

Data were pooled from both trial groups and presented for the whole cohort, adjusted for trial group. Participant characteristics were summarized at baseline and 1‐year follow‐up using mean (sd) values or frequencies. To examine differences in characteristics between participants who achieved remission and those who did not, we used the chi‐squared and *t*‐test as appropriate. We also examined differences between characteristics of participants with and without missing data; we assessed predictors of missing weight or remission information by comparing distributions of factors measured at baseline between those who were and were not missing weight or remission data. Percentage weight change over the first year after diagnosis was the main exposure variable. We also examined percentage weight change between 1 and 5 years. We constructed models using percentage weight change in four categories as follows: no weight change (reference category as ± 2.5%); weight gain (≥2.5%); ≤ 2.5–5% weight loss; ≤5–10% weight loss; and ≥10% weight loss. We also examined weight change in kilograms as a continuous variable. Change over the first year, and between 1 and 5 years in physical activity and diet, as continuous variables (daily intake of total energy, fat as a percentage of energy, fibre, alcohol and plasma vitamin C), were then examined. We constructed log binomial regression models to examine the association between change in these exposures and 5‐year risk of remission, generating risk ratios and 95% CIs. Multivariable nested models were then constructed adjusted on *a priori* reasoning. Model 1 was adjusted for baseline weight and follow‐up period. Model 2 was additionally adjusted for age, sex, ethnicity (white or other), education level (full‐time education finished at <16 years or >16 years, occupation (managerial and professional, intermediate and manual) trial group, date of diabetes diagnosis and clustering within practices. Given the possibility that remission might be more easily achieved amongst participants who had a lower HbA_1c_ level at baseline, we carried out a subgroup anlaysis that included only those with an HbA_1c_ level >48 mmol/mol. Statistical analysis was conducted in stata version 14 (Stata, College Station, TX, USA)

## Results

### Participant characteristics

Of the 867 participants at baseline, 730 (84%) had weight and HbA_1c_ measures at 5‐year follow‐up and were included in the analysis. The mean (sd) age of included participants was 61 (7) years. Most were men (61%) and white (97%). A total of 49% had continued in full‐time education after the age of 16 years and 43% reported unskilled or manual occupations. At the 5‐year follow‐up, 55% of participants had initiated hypoglycaemic medications. There were few differences between those with and without data on weight change and HbA_1c_. Compared with included participants, those with missing data were less likely to have stayed in education after age 16 years (15% and 11%, respectively).

Between baseline and 1‐year follow‐up, improvements were seen in the majority of health behaviours and cardiovascular disease risk factors. Baseline participant characteristics and changes over the first year are shown in Table 1, stratified according to remission status at 5 years. Diabetes remission was achieved in 257 participants (30%) in this cohort. Those who achieved remission were more likely to be men, smokers and to have remained in full‐time education after age 16 years.

**Table 1:** Participant characteristics in the ADDITION‐Cambridge cohort according to 5‐year diabetes remission status

Characteristic	Remission of diabetes				Non‐remission of diabetes			
	*n*	Baseline	*n*	1 year	*n*	Baseline	*n*	1 year
**Sociodemographic**								
Age, years	257	62 (6.9)	_	_	610	61 (7.3)	_	_
White ethnicity, *n* (%)	257	251 (97)	_	_	610	597 (96)	_	_
Men, *n* (%)	257	152 (59)	_	_	610	232 (38)	_	_
Social status, *n* (%)								
Professional	253	83 (33)	_	_	594	194 (33)	_	_
Education, *n* (%)								
Full‐time education Finished between 16 and 18 years	252	110 (44)	_	_	599	225 (38)		
**Clinical**								
BMI, kg/m^2^	255	33 (5.7)	228	31 (5.5)	607	34 (5.7)	505	33 (5.6)
Waist circumference, cm	257	111 (14.5)	228	107 (14.1)	607	112 (12.9)	505	109 (13.1)
Systolic blood pressure, mmHg	257	141 (19.4)	228	135 (18.1)	608	142 (20.5)	504	137 (18.7)
Total cholesterol, mmol/l	249	5.3 (1.1)	229	4.4 (0.9)	559	5.4 (1.2)	504	4.6 (1.0)
HbA_1c_	250		225		596		501	
mmol/mol		50 (9)		43 (5)		62 (14)		50 (7)
%		6.7 (1.2)		6.1 (0.7)		7.8 (1.8)		6.7 (0.9)
Previous stroke, *n* (%)	251	7 (2)	224	11 (5)	606	24 (4)	503	25 (5)
Previous myocardial infarction, *n* (%)	251	15 (6)	225	15 (7)	602	59 (10)	506	47 (9)
Anti‐hypertensive medication, *n* (%)	249	149 (59)	229	157 (68)	601	350 (65)	522	347 (59)
Lipid‐lowering medication, *n* (%)	148	40 (27)	131	84 (64)	609	147 (24)	507	322 (64)
**Health behaviour**								
Physical activity, net MET h/day	256	11.4 (8.2)	227	11.2 (7.5)	608	11.4 (7.2)	520	11.9 (7.6)
Alcohol intake, units/week	254	9 (12)	228	8 (11)	599	7 (11)	508	6 (11)
Current smoker, *n* (%)	256	38 (15)	229	27 (12)	610	119 (20)	515	84 (16)
Diet								
Total energy, kcal/day	254	1941 (654)	225	1710 (597)	601	1977 (739)	518	1720 (607)
Energy from fat, %	254	32 (6)	225	30 (7)	601	33 (6)	518	31 (6)
Fibre, g/day	254	17 (7)	225	19 (11)	601	17 (7)	518	19 (11)
Vitamin C intake, mg/day	254	133 (71)	225	142 (99)	601	128 (66)	518	139 (107)

Data are mean (sd), unless otherwise stated.

### Weight change and remission of diabetes at 5 years

In log binomial regression models, we observed that people who lost ≥ 10% body weight in the first year after diagnosis of diabetes were significantly more likely to achieve remission at 5 years compared to those with stable or increased weight. We observed similar trends with more modest weight loss of 5–10% or lower over the first year after diagnosis, but this was not statistically significant; these results are shown in Table 2. Similar associations were observed in the analysis of unit changes in weight, with strong positive correlations between 1‐kg weight loss and remission, as shown in Table 4. Similar trends were observed between 1 and 5 year follow‐up (Table 3). The greater the amount of weight loss achieved, the higher the likelihood of remission in all models (Table  5). In the subgroup analysis of participants with a baseline HbA_1c_ >48 mmol/mol, similar trends were observed between weight change and remission. This is shown in Tables S1 and S2.

**Table 2:** Association between percentage weight change category in the first year after diagnosis, and the risk of remission at 5 years in the ADDITION‐Cambridge study

	% weight change category	*n*		Risk ratio	95% CI		*p*
Unadjusted	No change (±2.5% change from baseline)	867	266	1			
	Weight gain (≥2.5%)		207	0.89	0.64	1.21	0.44
	Weight loss >2.5 to <5%		151	1.01	0.72	1.40	0.97
	Weight loss ≥5 to <10%		138	1.32	0.97	1.79	0.07
	Weight loss ≥10%		105	1.85	1.39	2.45	<0.01
Adjusted model 1*	No change (±2.5% change from baseline)	670	226	1			
	Weight gain (≥2.5%)		97	0.77	0.50	1.17	0.23
	Weight loss >2.5 to <5%		140	1.01	0.72	1.40	0.95
	Weight loss ≥5 to <10%		118	1.29	0.94	1.77	0.11
	Weight loss ≥10%		89	1.76	1.31	2.35	<0.01
Adjusted model 2†	No change (±2.5% change from baseline)	648	221	1			
	Weight gain (≥2.5%)		96	0.79	0.52	1.21	0.28
	Weight loss >2.5 to <5%		135	1.00	0.71	1.40	0.99
	Weight loss ≥5 to <10%		113	1.24	0.91	1.72	0.17
	Weight loss ≥10%		83	1.77	1.32	2.38	<0.01

^*^Model 1 adjusted for baseline weight and follow‐up period. ^†^ Model 2 adjusted for baseline weight, follow‐up period, age, sex, ethnicity, socio‐economic group, education level, occupation, trial group, clustering of practices and date of diabetes diagnosis

**Table 3:** Association between percentage weight change category between 1 and 5 years after diagnosis, and the risk of remission at 5 years in the ADDITION‐Cambridge study

	% weight change category	*n*		Risk ratio	95% CI		*p*
Unadjusted	No change (±2.5% change from baseline)	867	242	1			
	Weight gain (≥2.5%)		392	0.75	0.57	0.98	0.05
	Weight loss >2.5 to <5%		93	1.24	0.90	1.74	0.19
	Weight loss ≥5 to <10%		103	1.36	0.99	1.85	0.05
	Weight loss ≥10%		37	2.30	1.71	3.09	<0.01
Adjusted model 1*	No change (±2.5% change from baseline)	670	227	1			
	Weight gain (≥2.5%)		230	0.86	0.64	1.17	0.33
	Weight loss >2.5 to <5%		88	1.24	0.89	1.76	0.22
	Weight loss ≥5 to <10%		93	1.39	1.01	1.91	0.04
	Weight loss ≥10%		32	2.50	1.86	3.37	<0.01
Adjusted model 2†	No change (±2.5% change from baseline)	648	222	1			
	Weight gain (≥2.5%)		225	0.85	0.63	1.15	0.30
	Weight loss >2.5 to <5%		82	1.35	0.95	1.91	0.09
	Weight loss ≥5 to <10%		89	1.43	1.03	1.98	0.02
	Weight loss ≥10%		30	2.43	1.78	3.31	<0.01

* Model 1 adjusted for 1‐year weight and follow‐up period.

† Model 2 adjusted for 1‐year weight, follow‐up period, age, sex, ethnicity, socio‐economic group, education level, occupation, trial group, clustering of practices and date of diabetes diagnosis.

**Table 4:** Associations between change in weight and health behaviours in the first year after diagnosis and the risk of remission at 5 years

Variable	Unadjusted					model 1 *					model 2 †				
	*n*	Risk ratio	95% CI		*P*	*n*	Risk ratio	95% CI		*p*	*n*	Risk ratio	95% CI		*p*
Weight (kg)	730	1.06	1.03	1.09	<0.01	635	1.07	1.04	1.11	<0.01	632	1.07	1.04	1.11	<0.01
Physical activity (net MET h/day)	747	0.99	0.96	1.01	0.37	702	0.99	0.96	1.01	0.45	702	0.98	0.96	1.02	0.50
Alcohol intake (units/week)	726	1.00	0.98	1.03	0.65	683	1.03	1.00	1.07	0.04	683	1.04	1.00	1.08	0.04
Current smoker	744	0.72	0.30	1.73	0.47	699	0.69	0.23	1.89	0.50	699	0.69	0.23	2.01	0.50
Total energy (kcal/day)	736	1.00	0.99	1.00	0.44	692	1.00	0.99	1.00	0.38	692	1.00	0.99	1.00	0.38
Energy from fat (%)	736	1.00	0.98	1.03	0.47	692	1.00	0.97	1.02	0.91	692	0.99	0.97	1.02	0.91
Fibre (g/day)	736	1.00	0.99	1.01	0.79	692	1.00	0.97	1.03	0.90	692	1.00	0.97	1.03	0.90
Vitamin C intake (mg/day)	739	0.99	0.99	1.00	0.96	692	0.99	0.99	1.00	0.90	692	1.00	0.99	1.00	0.58

* Model 1 adjusted for baseline health behaviour and follow‐up period.

† Model 2 adjusted for baseline health behaviour, follow‐up period, age, sex, ethnicity, socio‐economic group, education level, occupation, trial group, clustering of practices and date of diabetes diagnosis.

**Table 5:** Associations between change in weight and health behaviours between 1 and 5 years after diagnosis and the risk of remission at 5 years

Variable	Unadjusted					model 1 *					model 2 †				
	*n*	Risk ratio	95% CI		*P*	*n*	Risk ratio	95% CI		*P*	*n*	Risk ratio	95% CI		*P*
Weight (kg)	680	1.06	1.03	1.09	<0.01	628	1.07	1.04	1.10	<0.01	607	1.08	1.04	1.11	<0.01
Physical activity (net MET h/day)	632	0.98	0.96	1.01	0.24	632	0.99	0.96	1.01	0.26	611	0.99	0.99	1.01	0.28
Alcohol intake (units/week)	635	0.99	0.96	1.01	0.48	626	0.98	0.95	1.00	0.18	606	0.98	0.95	1.01	0.24
Current smoker‡	643	0.70	0.35	1.40	0.38	643	0.73	0.35	1.53	0.41	621	0.77	0.36	1.63	0.49
Total energy (kcal/day)	623	1.00	0.99	1.00	0.73	619	1.00	0.99	1.00	0.72	599	1.00	0.99	1.00	0.59
Energy from fat (%)	623	1.00	0.97	1.02	0.84	619	1.00	0.97	1.02	0.86	599	0.99	0.99	1.02	0.92
Fibre (g/day)	623	1.00	0.99	1.02	0.31	623	1.00	0.98	1.02	0.82	602	1.00	0.98	1.02	0.72
Vitamin C intake (mg/day)	623	1.00	0.99	1.00	0.96	623	1.00	0.99	1.00	0.76	602	1.00	0.99	1.00	0.71

* Model 1 adjusted for baseline health behaviour and follow‐up period.

† Model 2 adjusted for baseline health behaviour, follow‐up period, age, sex, ethnicity, socio‐economic group, education level, occupation, trial group, clustering of practices and date of diabetes diagnosis.

‡ Refers to change in smoking status.

### Behaviour change and remission of diabetes at 5 years

We did not observe any consistent patterns of associations between unit changes in health behaviours and remission of diabetes. A positive association with remission was noted with unit changes in alcohol levels, but these varied between unadjusted and adjusted models, which are shown in Table 4.

## Discussion

In this prospective cohort study, we investigated the association between weight loss and remission of Type 2 diabetes at 5 years. We found that modest weight loss of ≥10% in the first year or first 5 years after diagnosis was strongly associated with remission of Type 2 diabetes. These findings suggest that remission is achievable without intensive lifestyle interventions or extreme calorie restrictions.

The present findings support and add to previous research that has demonstrated associations between weight loss and remission of Type 2 diabetes 5, 7, 18. For example, The DIRECT trial, also set in UK primary care, reported varying rates of remission of diabetes, depending on weight loss. The trial had aimed for a 15‐kg weight loss through an intensive intervention that included withdrawal of antidiabetic and anti‐hypertensive drugs, total diet replacement (825–853 kcal/day formula diet for 3–5 months), stepped food reintroduction (2–8 weeks), and structured support for long‐term weight loss maintenance. On average, 10 kg (15%) of weight was lost in the intervention group and half of the participants achieved remission. 7, 19. Other studies with similar intensive interventions in highly selected populations include the Counterbalance trial and the Look AHEAD trial. The Look AHEAD trial did not report remission as a primary outcome, but did include intensive support through dietary and physical activity programmes which resulted in remission. Whilst our observational findings are consistent with these trials, the specific amount of weight loss required to achieve remission varies. Most previous studies advocate significant weight loss (>15%), with the DIRECT, Counterbalance and Look AHEAD trials reporting between 5‐ and 20‐kg weight loss in order to achieve diabetes remission 5, 7. However, while baseline HbA_1c_ values were lower in our screen‐detected cohort, our results suggest that more modest weight loss of >10% is associated with a higher likelihood of remission if this occurs early in the disease trajectory. This may provide some rationale for motivating people with newly diagnosed Type 2 diabetes to lose weight rather than focusing on specific and potentially unachievable weight targets. Previous studies have shown that, when attempting to lose weight, people often set unrealistically high weight loss goals that could be detrimental to success, and evidence on whether weight loss counselling with specific targets is always successful is inconsistent 20. Indeed, the DIRECT trial did not manage to achieve the weight loss targets intended for most participants, with only 24% managing the 15‐kg target weight loss despite the intensive support. Furthermore, these interventions are unlikely to be scalable to the wider population because of their intensity and cost and the limited availability of facilitators 7. Our data suggest that, in addition to extending availability of intensive weight loss interventions, policymakers should consider a range of accessible approaches targeting weight loss amongst people with newly diagnosed diabetes.

Finally, while we observed associations between weight change and remission, we did not observe consistent associations between behaviour change and remission. This might be attributable to the differential precision of the exposure measurements, most of which were self‐reported and therefore subject to error and bias. For example, we found that self‐reported alcohol intake was associated with remission. Although there is some mixed evidence in the literature suggesting that moderate alcohol intake could be associated with positive cardiovascular outcomes, our findings were not consistent between adjusted and unadjusted models 21. It is therefore unlikely to be a true association. It could be due to chance or residual confounding.

The present study included people with Type 2 diabetes from a large population‐based sample across an extensive geographical area in the East of England in routine clinical follow‐up. We used measures of remission that are available in clinical practice to allow translation of our findings to practice. There was heterogeneity in this cohort with regard to socio‐economic groups, disease severity and health behaviours. There were also no specific dietary or physical activity restrictions for participants. This means that the study is generalizable to wider diabetes populations outside clinical trial cohorts; however, the sample was not ethnically diverse, comprising predominantly white European participants, which reflects the local population. Other strengths include the duration of follow‐up which was 5 years; most previous remission studies were of < 12 months’ duration. Also, participant retention in the present cohort was high, being 95% at 1‐year follow‐up and 83% at the 5‐year follow‐up. Behaviours were measured using previously validated questionnaires and repeated measures with the same instruments, reducing our concerns about measurement error. We did, however, conduct a number of hypothesis tests, so chance remains a plausible explanation for our findings.

In conclusion, remission of Type 2 diabetes is achievable in the longer term with modest weight loss of >10% early in the disease trajectory. This can be achieved without intensive interventions in free‐living populations. Our findings should inform discussions with people who have newly diagnosed Type 2 diabetes as motivation towards remission of the disease without restrictive and sometimes unachievable calorie restrictions. Further work is needed to replicate these findings in more ethnically and socially diverse populations. Further examination will need to include an assessment of the relationship between remission and longer‐term clinical outcomes, such as mortality.

## Funding sources

ADDITION‐Cambridge was supported by the Wellcome Trust (grant reference no: G061895), the Medical Research Council (grant reference no: G0001164 and Epidemiology Unit programme: MC_UU_12015/4), the National Institute for Health Research (NIHR) Health Technology Assessment Programme (grant reference no: 08/116/300), NIHR Programme Grants for Applied Research (RP‐PG‐0606‐1259) National Health Service R&D support funding (including the Primary Care Research and Diabetes Research Networks) and the NIHR. S.J.G. is an NIHR Senior Investigator. The University of Cambridge has received salary support in respect of S.J.G. from the NHS in the East of England through the Clinical Academic Reserve. Bio‐Rad provided equipment for HbA_1c_ testing during the screening phase. The Primary Care Unit is a member of the NIHR School for Primary Care Research and supported by NIHR Research funds. G.I. was an NIHR Clinical Lecturer. H.D.M. was an NIHR Doctoral Research Fellow at the time of this study and is now an NIHR Clinical Lecturer. J.S. is supported by an MRC Epidemiology Unit Core programme MC_UU_12015/4 fellowship. The views expressed are those of the author(s) and not necessarily those of the NHS, the NIHR or the Department of Health and Social Care. The sponsor had no role in study data collection, data analysis, data interpretation, or writing of the findings. The corresponding author had full access to all the data in the study and had final responsibility for the decision to submit for publication.

## Competing interests

S.J.G. reports grants from the Wellcome Trust, Medical Research Council, NIHR, NIHR Health Technology Assessment Programme, NHS R&D and the University of Aarhus (Denmark), and provision of equipment from Bio‐Rad during the conduct of the study. Outside the submitted work he also reports receiving fees from Novo Nordisk, Astra Zeneca and Napp for speaking at postgraduate education meetings, support to attend a scientific meeting from Napp, and an honorarium and reimbursement of travel expenses from Eli Lilly, associated with membership of an independent data monitoring committee for a randomized trial of a medication to lower glucose.

## Supporting information


**Table S1.** Association between percentage weight change category in the first year after diagnosis, and the risk of remission at five‐years in the ADDITION‐Cambridge study amongst participants with HbA_1c_ > 6.5%.
**Table S2.** Association between percentage weight change category between 1 to 5 years after diagnosis, and the risk of remission at five‐years in the ADDITION‐Cambridge study amongst participants with HbA_1c_ > 6.5%.
