# Supplementary material for: Behaviour change, weight loss and remission of Type 2 diabetes: a community‐based prospective cohort study
Source: Diabet Med. 2019 Sep 26;37(4):681–8. doi: 10.1111/dme.14122 (PMC7155116; doi:10.1111/dme.14122)
Supplement: Supplementary file 1 — Table S1. Association between percentage weight change category in the first year after diagnosis, and the risk of remission at five‐years in the ADDITION‐Cambridge study amongst participants with HbA1c > 6.5%. Table S2. Association between percentage weight change category between 1 to 5 years after diagnosis, and the risk of remission at five‐years in the ADDITION‐Cambridge study amongst participants with HbA1c > 6.5%. [file DME-37-681-s001.docx]

Supplementary tables:

**Table S1. Association between percentage weight change category in the first year after diagnosis, and the risk of remission at five-years in the ADDITION-Cambridge study amongst participants with HbA_1c_ > 6.5%**

|  | % weight change category | n | risk ratio | 95% CI | | P-value |
| --- | --- | --- | --- | --- | --- | --- |
| unadjusted | no change (±2.5% change from baseline) | 561 | 1 |  |  |  |
|  | weight gain (≥2.5%) |  | 0.67 | 0.39 | 1.13 | 0.13 |
|  | weight loss >2.5-<5% |  | 0.93 | 0.57 | 1.53 | 0.78 |
|  | weight loss ≥5-<10% |  | 1.49 | 0.96 | 2.30 | 0.07 |
|  | weight loss ≥10% |  | 1.86 | 1.20 | 2.87 | 0.01 |
| adjusted model 1* | no change (±2.5% change from baseline) | 429 | 1 |  |  |  |
|  | weight gain (≥2.5%) |  | 0.54 | 0.27 | 1.11 | 0.10 |
|  | weight loss >2.5-<5% |  | 0.89 | 0.53 | 1.43 | 0.60 |
|  | weight loss ≥5-<10% |  | 1.30 | 0.81 | 2.06 | 0.26 |
|  | weight loss ≥10% |  | 1.60 | 1.01 | 2.51 | 0.04 |
|  |  |  |  |  |  |  |
| adjusted model 2** | no change (±2.5% change from baseline) | 413 | 1 |  |  |  |
|  | weight gain (≥2.5%) |  | 0.56 | 0.28 | 1.14 | 0.11 |
|  | weight loss >2.5-<5% |  | 0.88 | 0.53 | 1.44 | 0.59 |
|  | weight loss ≥5-<10% |  | 1.26 | 0.77 | 2.05 | 0.36 |
|  | weight loss ≥10% |  | 1.65 | 1.04 | 2.63 | 0.03 |

**Table S2 Association between percentage weight change category between one to five years after diagnosis, and the risk of remission at five-years in the ADDITION-Cambridge study amongst participants with HbA_1c_ > 6.5%**

|  | % weight change category | n | risk ratio | 95% CI | | P-value |
| --- | --- | --- | --- | --- | --- | --- |
| unadjusted | no change (±2.5% change from baseline) | 561 | 1 |  |  |  |
|  | weight gain (≥2.5%) |  | 0.65 | 0.41 | 1.04 | 0.07 |
|  | weight loss >2.5-<<5% |  | 1.69 | 1.04 | 2.74 | 0.03 |
|  | weight loss ≥5-<10% |  | 1.77 | 1.10 | 2.84 | 0.01 |
|  | weight loss ≥10% |  | 3.48 | 2.29 | 5.30 | <0.01 |
|  |  |  |  |  |  |  |
| adjusted model 1* | no change (±2.5% change from baseline) | 429 |  |  |  |  |
|  | weight gain (≥2.5%) |  | 0.81 | 0.48 | 1.39 | 0.45 |
|  | weight loss >2.5-<5% |  | 1.76 | 1.06 | 2.93 | 0.03 |
|  | weight loss ≥5-<10% |  | 1.88 | 1.16 | 3.06 | 0.01 |
|  | weight loss ≥10% |  | 3.80 | 2.50 | 5.89 | <0.01 |
| adjusted model 2** | no change (±2.5% change from baseline) | 413 |  |  |  |  |
|  | weight gain (≥2.5%) |  | 0.76 | 0.44 | 1.30 | 0.32 |
|  | weight loss >2.5-<5% |  | 1.91 | 1.14 | 3.22 | 0.02 |
|  | weight loss ≥5-<10% |  | 1.88 | 1.15 | 3.08 | 0.01 |
|  | weight loss ≥10% |  | 3.99 | 2.59 | 6.16 | <0.01 |
